# Molecular Changes in Relation to Alcohol Consumption and Hepatocellular Carcinoma

**DOI:** 10.3390/ijms23179679

**Published:** 2022-08-26

**Authors:** Reina Sasaki-Tanaka, Ranjit Ray, Mitsuhiko Moriyama, Ratna B. Ray, Tatsuo Kanda

**Affiliations:** 1 Division of Gastroenterology and Hepatology, Department of Medicine, Nihon University School of Medicine, 30-1 Oyaguchi-kamicho, Itabashi-ku, Tokyo 173-8610, Japan; 2 Departments of Internal Medicine, and Molecular Microbiology and Immunology, Saint Louis University, Saint Louis, MO 63104, USA; 3 Department of Pathology, Saint Louis University, Saint Louis, MO 63104, USA

**Keywords:** hepatocellular carcinoma, alcohol, acetaldehyde, ALDH, cirrhosis

## Abstract

Alcohol is the one of the major causes of liver diseases and promotes liver cirrhosis and hepatocellular carcinoma (HCC). In hepatocytes, alcohol is converted to acetaldehyde, which causes hepatic steatosis, cellular apoptosis, endoplasmic reticulum stress, peroxidation, production of cytokines and reduces immune surveillance. Endotoxin and lipopolysaccharide produced from intestinal bacteria also enhance the production of cytokines. The development of hepatic fibrosis and the occurrence of HCC are induced by these alcohol metabolites. Several host genetic factors have recently been identified in this process. Here, we reviewed the molecular mechanism associated with HCC in alcoholic liver disease.

## 1. Introduction

Hepatocellular carcinoma (HCC) is a serious problem in chronic liver diseases and cirrhosis, which are associated with chronic hepatitis B virus (HBV) and hepatitis C virus (HCV) infection [1], and non-alcoholic steatohepatitis (NASH) [2,3]. It is also well-known that alcohol consumption causes hepatic steatosis, hepatic fibrosis, cirrhosis and HCC in patients with or without HCV infection [4]. Globally, alcoholic liver disease is a major health problem.

HCC is associated with chronic hepatic inflammation and hepatic fibrosis derived from various liver diseases with different etiologies [1,2,3,4]. There are some common molecular pathways, which are involved in different etiology of HCC [2,3,5,6]. Some specific molecular mechanisms are known in the incidence of HCC among patients with alcoholic liver diseases. The quantity of alcohol consumption for developing alcoholic hepatitis is not clear.

The lower limit of daily alcohol use for the development of cirrhosis is in the range of 30–50 g of ethanol [7]. However, most patients with alcoholic hepatitis drink more than 100 g daily [8]. In general, most patients with alcoholic liver disease reported heavy alcohol use (more than 100 g daily) for two or more decades [7]. Overweight subjects for at least 10 years are additional risk factor for cirrhosis, acute alcoholic hepatitis and steatosis [8]. Patients with NAFLD or NASH have a negative history of alcohol abuse, as indicated by a weekly ethanol use of <140 g in women, and <210 g in men. Alcoholic beverages may be responsible for steatosis but also for progression of nonalcoholic fatty liver disease (NAFLD) to NASH and/or cirrhosis [9]. Moderate alcohol consumption is negatively related to the incidence of NASH and liver fibrosis; moderate alcohol consumption is positively linked with the incidence of HCC in patients with chronic viral liver diseases [10].

Current therapy is effective in early stages of HCC. Understanding the molecular mechanism is essential for effective and targeted therapy for HCC. Immune checkpoint inhibitors are approved for HCC treatment. The combination of targeted therapy molecules and immune modulators can enhance the efficacy of the therapy. In this article, we reviewed the molecular mechanisms for HCC occurrence from alcoholic liver disease. It is important to understand the molecular changes in relation to alcohol consumption and HCC. These could provide the early detection and early treatment of HCC associated with alcoholic liver diseases, resulting in the improvement of prognosis of patients with HCC-related alcoholic liver diseases.

## 2. Alcohol Metabolism and Liver Injuries

### 2.1. Alcohol Oxidation

**Figure 1:**
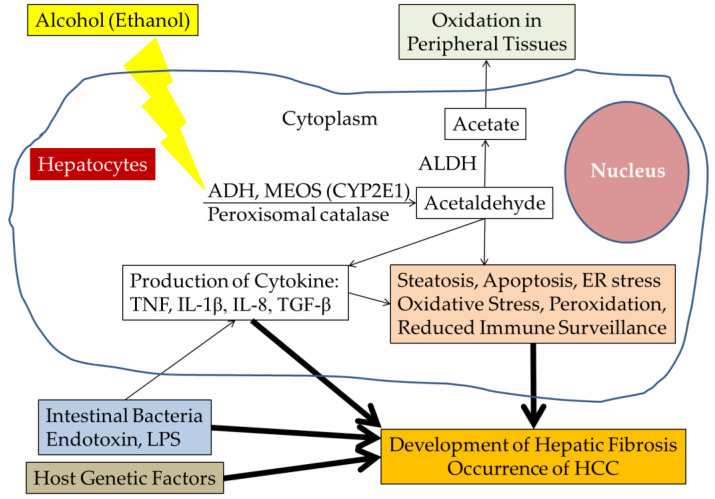
Schematic presentation depicting potential steps leading to liver pathogenesis from alcohol abuse. ALDH, aldehyde dehydrogenase; ADH, alcohol dehydrogenase; MEOS, microsomal ethanol-oxidizing system; CYP2E1, cytochrome P450IIE1; TNF, tumor necrosis factor; IL-1ß, interleukin-1ß; IL-8, interleukin-8; TGF-ß, transforming growth factor β1; ER, endoplasmic reticulum; LPS, lipopolysaccharide; HCC, hepatocellular carcinoma.

Alcohol includes ethanol, which is oxidized mainly in the liver (Figure 1) [11]. Ethanol is also oxidized less in the gastrointestinal tract than the liver [11]. Alcohol dehydrogenase (ADH) catabolizes ~80% ethanol oxidation by initial conversion to acetaldehyde in the cytosol of hepatocytes [12,13]. The resulting increased ratio of reduced nicotinamide adenine dinucleotide (NADH)/oxidized nicotinamide adenine dinucleotide (NAD), derived from the vitamin niacin, ref. [14] may play a role in the initial pathogenesis of alcohol-induced steatosis [15]. Dietary nicotinic acid supplementation is reported to improve chronic alcohol-induced fatty liver in rats [16].

The microsomal ethanol-oxidizing system (MEOS) pathway metabolizes most of the remaining ethanol [12,13,15,16]. Cytochrome P450IIE1 (CYP2E1) is involved in this MEOS pathway. Although hepatic injury in alcoholics due to intake of acetaminophen has been reported, susceptibility to acetaminophen hepatotoxicity is apparently caused by the induction of CYP2E1 by ethanol and by depletion of glutathione (GSH) [17]. A minor proportion of ethanol is also metabolized through the peroxisome catalase-dependent pathway [12,18]. The ADH pathway may be a major pathway in the conversion of ethanol to acetaldehyde in the liver [18]. Aldehyde dehydrogenases (ALDHs) further oxidize most of acetaldehyde derived from ethanol to acetate in the liver. Acetaldehyde accumulation may account for the rinsing cause with alcohol consumption in patients with an inactive form of ALDH [19].

Hepatotoxicity, resulting from the CYP2E1-mediated activation of acetaminophen, was exhibited in the livers of human CYP2E1-transgenic mice by elevated serum alanine aminotransferase (ALT) levels, enhanced hepatocyte necrosis, and decreased CYP2E1 levels [20]. Alcoholic liver disease was enhanced in CYP2E1-transgenic mice [21]. CYP2E1 may play a role in alcohol-enhanced steatohepatitis [22] and potentiate TNF-induced liver injury [23]. Autophagy also demonstrates a protective effect on CYP2E1-dependent liver injury after chronic ethanol treatment [23].

### 2.2. Acetaldehyde

Acetaldehyde may have toxic effects on the liver. The accumulation of acetaldehyde facilitates the progression of alcoholic fatty liver and other liver diseases, including non-alcoholic fatty liver disease (NAFLD), viral hepatitis and HCC, through adduct formation and inflammatory responses [24]. The accumulation of acetaldehyde increases the sensitivity to tumor necrosis factor (TNF)-mediated hepatocyte cell death [12,25,26,27,28].

Acetaldehyde increases TNF-α and interleukin (IL)-8 expression, stimulates IL-1β and IL-8 secretion, increases lipid peroxidation damage and decreases catalase activity in human hepatoma HepG2 cells [25]. Apoptosis caused by chronic ethanol treatment may be due to the potentiation of TNF-induced p38 mitogen activated protein kinase (MAPK) [26]. A major source of the surge in circulating proinflammatory cytokines is Kupffer cells, and they are sensitized to generate TNF-α [27]. The regulation of autophagy is also involved in the formation of steatosis and inflammation in chronic alcohol intake-patients [28].

Acetaldehyde binds to host proteins, induces of endoplasmic reticulum (ER) stress and forms of neoantigen [12,29,30,31]. In mice, acetaldehyde dehydrogenase 2 (ALDH2) increases the expression of glucose-regulated protein 78 (GRP78) and ER stress-related protein phosphorylated eukaryotic initiation factor 2 (peIF2α). ALDH2 decreases the expression of apoptosis-related protein, including C/EBP homologous protein (CHOP), caspase 12 and caspase 9, resulting in the induction of ER stress response and apoptosis [29]. Acetaldehyde also suppresses HBV-major histocompatibility complex (MHC) class I associated antigen presentation on hepatocytes by induction of ER stress and Golgi fragmentation [30]. Ethanol and its metabolites, acetaldehyde and fatty acid ethyl esters, cause cytotoxicity, ER/oxidative and mitochondrial stress, and dysregulate AMP-activated protein kinase α (AMPKα) signaling [31].

### 2.3. Hepatic Steatosis, Oxidative Stress and Peroxidation

The pathology of the liver from NAFLD and NASH mimics alcoholic steatosis and alcoholic liver disease [2]. Steatosis is a prominent feature of alcoholic liver disease. As described above, alcohol oxidation leads to fat deposition in the hepatocytes [12]. Alcoholic liver disease includes hepatic steatosis, oxidative stress, acetaldehyde-mediated toxicity, and cytokine/chemokine-induced inflammation [32].

The increased ratio of NADH/NAD in hepatocytes disrupts mitochondrial β-oxidation of fatty acids and results in steatosis of liver in patients with alcohol consumption [33]. Alcohol consumption can directly activate sterol regulatory element-binding protein 1c (SREBP-1c) and inactivate the expression of peroxisome proliferator-activated receptor (PPAR)-α, leading to the induction of fatty acid synthesis and inhibition of fatty liver β-oxidation, which leads to the development of alcoholic steatosis [33,34,35,36]. Alcohol exposure also inhibits AMPK [37] and, subsequently, increases acetyl-CoA carboxylase (ACC) activity [38], but decreases carnitine palmitoyl transferase 1 (CPT-1) activity [39], leading to an increase in fatty acid synthesis and a decrease in fatty acid β-oxidation [33].

## 3. Immune Mechanism of Alcoholic Liver Diseases

### 3.1. Endotoxin

There is no animal model that fully mirrors human alcoholic liver disease by alcohol consumption, as rodents have a natural aversion for alcohol consumption [40]. Inflammatory activation of resident Kupffer cells by portal-derived lipopolysaccharide (LPS) has a leading role for alcoholic liver disease in animal models [41]. Endotoxin, which is also known as LPS, is produced by intestinal bacteria. Alcohol consumption increases endotoxin level in liver and blood, enhances inflammatory cytokine production by Kupffer cells, and the generation of reactive oxygen species (ROS) in liver for damaging hepatocytes [42,43]. ROS accumulation generates structural and functional changes in the DNA for cell cycle change leading to carcinogenesis [44]. Oxidative stress induces lipid peroxidation products which enhance the actions of endotoxins by gut bacteria [45], and induces mutations in the p53 gene, which develops HCC [46].

Neutrophils not only promote hepatocyte injury by producing ROS but may also help in liver repair by removing apoptotic or necrotic hepatocytes and produce growth factors. Neutrophils play an important role in controlling bacterial infection in alcoholic liver disease, but severe alcoholic liver disease is combined with impaired phagocytic and bactericidal activities [47,48,49,50]. Thus, similar phenomena which observed in the Kupffer cells are also observed in neutrophils, with the consequence of recruitment of neutrophils. Intestinal CYP2E1 promotes gut LPS transporting into blood through the alcohol-induced increased gut leakage, resulting in endotoxemia and steatosis and steatohepatitis of the liver [23].

### 3.2. Innate Immunity and Adaptive Immunity

The development of alcoholic liver disease also impairs innate and adaptive immune system [51]. Complement activation plays a role in developing alcoholic liver disease. Classical complement pathway can be activated by complement component 1q (C1q) binding to apoptotic cells in liver for hepatic inflammation [51,52]. The complement component 3 (C3) contributes triglyceride accumulation in the liver and adipose tissues, while complement component 5 (C5) involves in inflammation and injury for chronic ethanol consumption [51,53,54].

Ethanol exposure activates both cellular and circulating innate immune components [55]. Ethanol exposure increases toll-like receptor-4 (TLR4)-dependent cytokine expression in Kupffer cells and redox signaling dysregulation [55,56]. Conversely, ethanol suppresses innate immune responses impairing TLR3 signaling mechanism, various innate effector molecules, and proinflammatory cytokines and chemokines [57].

Alcohol-dependent subjects with altered intestinal permeability change gut-microbiota composition [58]. Alcohol causes intestinal dysbiosis, resulting in the reduction of the capacity of the microbiome to synthesize saturated long-chain fatty acids and the proportion of Lactobacillus species [59]. Gut-associated bacteria contribute to the pathogenesis of alcoholic liver diseases by their structural components (pathogen-associated molecular patterns, PAMPs), like TLR4-ligand LPS, or by their metabolites altering gut mucosal integrity [60]. A host of bacterial PAMPs activate the cells of innate immune systems via binding to specific receptors, including TLRs in cells [60]. Cluster of differentiation 14 (CD14) and myeloid differentiation protein-2 (MD-2), bind LPS and upon LPS-binding activate TLR4 [61,62]. Gut-derived LPS activates hepatic macrophages via these molecules.

LPS-induced TNF-α plays a key role in the development of alcoholic liver disease [60]. Patients with alcoholic liver disease have increased serum levels of IL-1, TNF-α and IL-8, elevated expression of caspase-1 and nucleotide-binding oligomerization domain-like receptor (NLR) family pyrin domain-containing 3 (NLRP3), neutrophilia, and activation of monocytes and macrophages in the liver [60]. The role of inflammasomal activation has been also confirmed in alcoholic liver disease [63].

Alcohol dysregulates function of lymphocytes, neutrophils, monocytes, and tissue macrophages of the innate immune system and these eventually adaptive immune responses [64]. Circulating immunoglobulin A (IgA) is elevated in patients with alcoholic liver disease [65,66]. As T cells generally control IgA production produced by B cells, higher level of IgA suggests T cell independent drive to IgA production exists in alcoholic cirrhosis [67] In patients with alcoholic liver diseases, inflammation-induced liver-resident IgA-producing cells accumulate in fibrotic liver, and may dismantle anti-liver cancer immunity [68]. Liver-resident IgA-producing cells express programmed death ligand 1 (PD-L1) and IL-10, and directly induce exhaustion of liver cytotoxic CD8+ T-lymphocytes. These events prevent the appearance of HCC and the expression of a limited repertoire of T-cell receptors against tumor-associated antigens in HCC development [69]. Gut microbes may also promote HCC development and liver-resident IgA-producing plasmocytes may suppress CTL activation [69]. Thus, adaptive immune response is also impaired in patients with alcoholic liver disease. As PD-L1 blockade induces HCC regression, the analysis of these mechanism may be useful for the further development of HCC treatment [69,70].

### 3.3. Chemokines

The levels of circulating chemokine (C-X-C motif) ligand 8 (CXCL8: or IL-8) were highly elevated in patients with alcoholic hepatitis, particularly in those who died [71,72]. Different CXC chemokines, particularly CXCL8, mediates recruitment of neutrophils, which contribute to liver tissue damage in the alcoholic liver disease [73]. CXCL8 also exacerbates alcohol-induced fatty liver diseases in mice [74]. CCL20, which is a ligand of CCR6, mediates hepatic inflammation, promotes hepatic fibrogenesis and mediates the effects of LPS on liver injury [75].

Activated macrophages are divided into M1 macrophages and M2 macrophages, and both are closely associated to inflammatory responses. M1 macrophages are mainly involved in pro-inflammatory responses, whereas M2 macrophages are mainly involved in anti-inflammatory responses. Among CC chemokines, CCL2 also promotes inflammation, fibrosis, and possibly steatosis [76]. The expression of CCL2 in alcoholic liver disease is associated with disease severity and neutrophil infiltrates in the liver [77]. Thus, similar to various cytokines, chemokines also play pivotal roles in the pathogenesis of alcoholic liver disease [71].

## 4. Alcohol and Hepatic Fibrosis

### Possible Host Factors Affecting Hepatic Fibrosis in Alcoholic Liver Diseases

Alcohol is a common cause of cirrhosis [12]. Women seem to progress from alcoholic hepatitis to cirrhosis even if they withdraw from alcohol [12,68]. Estriol treatment in rats enhances LPS-induced nitric oxide (NO) production in Kupffer cells [68]. Male sex hormones may protect the liver against ethanol-related damage [78]. The androgen receptor may also be involved in the development of disease progression in chronic hepatitis C [79,80].

A recent, genome-wide, association study for alcohol-related cirrhosis identified risk loci in the mitochondrial amidoxime reducing component 1 gene (MARC1) and the heterogeneous nuclear ribonucleoprotein U-like 1 gene (HNRNPUL1) [81]. Minor A allele of MARC1:rs2642438 and minor C allele of HNRNPUL1:rs15052 were associated with reduced risk and increased risk, respectively, of alcohol-related cirrhosis (adjusted odds ratio, 0.76; *p* = 0.0027 and 1.30; *p* = 0.020) [81]. Single nucleotide polymorphism (SNP) rs35652124 variation in the transcription factor nuclear factor erythroid-related factor 2 (Nrf2)-encoding gene nuclear factor erythroid 2-related factor 2 (NFE2L2) is a potential genetic marker for susceptibility to alcoholic cirrhosis [82]. Variants of transmembrane 6 superfamily member 2 (TM6SF2) and membrane-bound O-acyltransferase domain-containing protein 7 (MBOAT7) gene polymorphisms are identified as risk factors for alcoholic liver cirrhosis (*p* = 7.89 × 10^−10^ and *p* = 1.03 × 10^−9^, respectively) [83]. Patatin-like phospholipase domain containing 3 (PNPLA3) rs738409 is also an important risk locus for alcohol-related cirrhosis (*p* = 1.54 × 10^−48^) at a genome-wide level of significance [83]. PNPLA3 is also involved in liver fibrosis in patients with NASH/NAFLD [84,85,86]. Of interest, these three loci play a role in lipid processing. Thus, several genetic factors could be associated with the development of alcoholic liver fibrosis.

Hepatic fibrosis accompanies an accumulation of extracellular matrix (ECM) from activation of hepatic stellate cells (HSCs) and the production of transforming growth factor β1 (TGF-β1) [87]. HSCs are the primary source of activated myofibroblasts that produce ECM in the liver [88]. HSCs are a major fibrogenic cell type in the liver and contribute to collagen accumulation during chronic liver disease [89]. In alcoholic liver disease, the additional effects of acetaldehyde, endotoxin, impairment of innate and adaptive immunity, and others on these pathways seem to enhance hepatic fibrosis. HSCs may be activated by enhanced cytokines in patients with alcoholic liver disease, leading to fibrosis [90]. Epigenetic mechanism, such as microRNA, may also be critical [91].

TGF-β is a potent inducer of fibrogenic epithelial-to-mesenchymal transitions (EMTs) [92]. TGF-β depends on RAS and MAPK pathway inputs for the induction of EMTs. RAS-responsive element binding protein 1 (RREB1), a RAS transcriptional effector [93], is a key partner of TGF-β-activated SMAD transcription factors in EMT. RREB1 provides a molecular link between RAS and TGF-β pathways for coordinated induction of developmental and fibrogenic EMTs. Transformed hepatocytes secreting TGF-β may also induce a cellular microenvironment to promote fibroblast activation for stromal changes [92]. These molecules may play roles in development of alcoholic fibrosis and cancer.

Interferon (IFN)-γ next to IFN-α, plays an important role in mechanisms of liver injury [94]. IFN-γ can induce IFN-α expression in mononuclear phagocytes (MNPs) [95]. IFN-γ reduces platelet endothelial-cell adhesion molecule (PECAM)-1 expression in parallel with the increase in intercellular cell adhesion molecule-1 (ICAM-1) expression. Early downregulation of PECAM-1 in parallel to an ICAM-1 enhancement is a likely important process for the adhesion and transmigration of inflammatory cells. TGF-β induced PECAM-1 up-regulation may be of importance during the recovery phase. Thus, TGF-β also works as a mediator of repair process after liver injury [96].

## 5. Alcohol and HCC

HCC is one of the male-dominant diseases, irrespective of etiology [78,97]. A meta-analysis of the dose-response relations for the amount of alcohol consumption and risk of HCC demonstrated a 1.8-times increase in risk for the heaviest drinkers (with more than 100 g daily), although alcohol consumption is one of the major strong risk factors for liver cirrhosis with a 27-times increase in risk on drinking 100 g daily [98,99,100,101].

In patients with alcoholic liver disease, the proportion of a loss of function variant (rs72613567) in 17-beta-hydroxysteroid dehydrogenase 13 (HSD17B13) carriers with HCC was significantly lower (32%) than in chronic liver disease patients without HCC (40%) [102]. PNPLA3-rs738409 and TM6SF2-rs58542926 are inherited risk variants of HCC development in patients with alcoholic liver disease [103]. Genetic polymorphism of ALDH 2 analysis [45] demonstrated that all HCC associated with pure alcoholic liver disease had ALDH 2(1)/2(1) in Japan [104].

Alcohol consumption is associated with other cancer risks than HCC [105]. Heavy drinkers with the ALDH2*2 allele are associated with poor survival in hypopharyngeal squamous cell carcinoma [106]. Both alcohol consumption level and ADH1C (rs698) and ALDH2 (rs671) polymorphisms are important to consider as gastric cancer risks [107]. The extent of the effect of the ADH polymorphisms is greater in subjects who were heavy drinkers, heavy smokers, and had esophageal cancer [108]. Drinking may also increase the risk of lung cancer, especially among individuals who have the variant ALDH (2) alleles [109].

Alcoholic liver diseases occasionally resemble autoimmune hepatitis or drug-induced liver injury. In such cases, it may be difficult to diagnose alcoholic liver disease, even from liver biopsy. Attention should be paid to the fact that alcoholic liver injury in some patients occurs with alcohol dependence [110].

## 6. Prevention and Treatment

**Figure 2:**
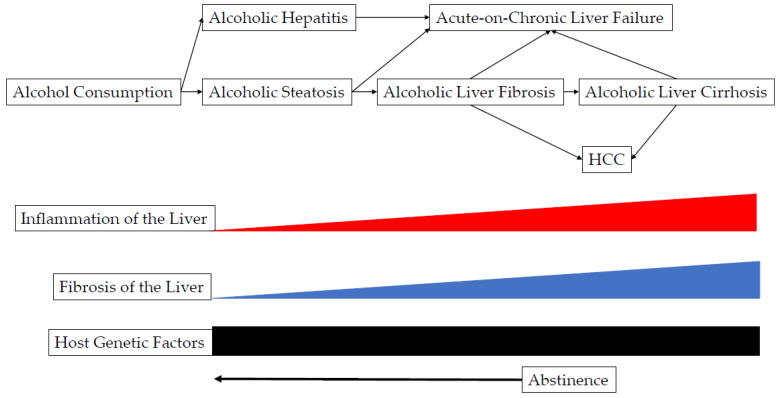
Molecular mechanisms: the connections between alcohol consumption and hepatocellular carcinoma.

Abstinence, i.e., stopping drinking is the best treatment for alcoholic liver diseases (Figure 2). Nalmefene is a μ opioid antagonist and a partial agonist at the κ receptor, as needed use may prevent the development of HCC in patients with alcoholic liver diseases [111,112].

Alcoholic liver disease is a major health problem. In this article, we discussed the molecular mechanisms of the occurrence of HCC induced by alcohol. The development of alcoholic liver disease includes genetic factors and gene polymorphism, along with alcohol consumption. On top of them, behavior and low socioeconomic status may enhance the risk factors for alcoholic liver disease. Alcoholic beverages induce not only the apoptosis of hepatocytes with the consequence of reduction of protein synthesis, but also the production of cytokines and chemokines. DNA damage by acetaldehyde, production of NO, and reduced immune surveillance may be associated with hepatocarcinogenesis (Figure 1). Another important area of HCC research is the identification of risk-predictive biomarkers. The prospective evaluation has been the major challenge because of long-term follow-up required for longitudinal group to observe the interruption of clinical outcomes and statistical correlation. Understanding the pathogenesis at the molecular level including genetic and epigenetic modifiers will help in developing new therapeutic targets.

## 7. Conclusions

Several studies are undergoing for the development of promising biomarkers and precision medicine and, hopefully, these will provide significant improvement in the poor prognosis of patients with alcoholic liver disease and HCC in the near future. Further, life-style changes for diet and alcohol consumption remain an important issue for controlling the surge of alcoholic liver disease.

## Data Availability

Not applicable.
